# p53 and energy balance: meeting hypothalamic AgRP neurons

**DOI:** 10.15698/cst2018.11.165

**Published:** 2018-10-18

**Authors:** Omar Al-Massadi, Mar Quiñones, Ruben Nogueiras

**Affiliations:** 1 Department of Physiology, CIMUS, University of Santiago de Compostela-Instituto de Investigación Sanitaria, Santiago de Compostela, 15782, Spain.; 2 CIBER Fisiopatología de la Obesidad y Nutrición (CIBERobn), 15706, Spain.

**Keywords:** p53, hypothalamus, obesity, energy expenditure

## Abstract

Cancer cells feature strong metabolic changes to cope with the high energy demand for cell growth and division. Given the importance of metabolic reprogramming in tumor development, it seems logical that tumor suppressors and oncogenes are also regulating the molecular pathways controlling these processes. The p53 tumor suppressor gene has been extensively studied for its role in responding to DNA damage, hypoxia, and oncogenic activation. During the last years, we have learnt that p53 has also the capacity to modulate metabolic changes in cells by regulating a large variety of pathways such as glycolysis, oxidative phosphorylation or fatty acid metabolism. Our group has recently found that the lack of p53 in AgRP neurons, but not POMC neurons, causes that mice are more prone to develop diet-induced obesity (Nat Commun. 9(1):3432). The reason for this is that these mice showed a late increase in food intake and especially because they had a reduced thermogenic activity in BAT. The mechanism modulating these actions involves the upregulation of MKK7 that activates c-Jun N-terminal kinase.

Cancer cells undergo profound metabolic changes to cope with the high energy demand for cell growth and division. Given the importance of this metabolic reprogramming in tumor development, it seems logical that tumor suppressors and oncogenes are also regulating the molecular pathways controlling these processes. The p53 tumor suppressor gene has been extensively studied for its role in responding to DNA damage, hypoxia, and oncogenic activation. In addition to these "classical" responses, p53 levels are also increased in the liver or white adipose tissue when mice are fed a high fat diet. A recent study of our laboratory has found that protein levels of p53 are increased in the hypothalamus of diet-induced obese animals. The mechanisms responsible for the activation of p53 by metabolic and other stress signals are complex, but once activated, p53 primarily exerts its functions by acting as a transcription factor, regulating the expression of both genes and microRNAs. During the last years, we have learnt that p53 has also the capacity to modulate metabolic changes in cells by regulating a large variety of pathways such as glycolysis, oxidative phosphorylation or fatty acid metabolism.

The functions of p53 to control various aspects of cellular metabolism lead to important physiological changes as reflected by different reports targeting p53 in different key metabolic organs. For instance, the depletion of p53 in white adipose tissue enhanced insulin sensitivity and glucose tolerance when mice were fed a high fat high sucrose diet, while its overexpression impairer both insulin sensitivity and glucose tolerance. In brown adipose tissue (BAT), the effects of p53 depend on when it is manipulated. Mice lacking p53 in BAT at embryonic stages did not show significant alterations in energy homeostasis. However, the knockdown of p53 in the BAT of adult mice slightly increased body weight and inhibited BAT thermogenic activity, while the overexpression of p53 in the BAT of adult mice reduced weight gain and stimulated BAT activity. In the liver, two studies have demonstrated that the lack of hepatic p53 triggers lipid accumulation, and these effects seem to be mediated by increased levels of p63, and stimulation of inhibitor of nuclear factor kappa beta (IKKβ) and endoplasmic reticulum stress. Finally, related to a pancreatic function, mice lacking p53 in the whole body were protected against the development of diabetes in streptozotocin-induced type 1 diabetes and db/db mouse models of type 2 diabetes.

In addition to the metabolic function of p53 in these peripheral organs, the ability of p53 to regulate energy homeostasis through the central nervous system has been recently deciphered. The central nervous system, and particularly the hypothalamus, orchestrates the regulation of many different biological actions including energy balance. This requires that the brain receives continuous information about energy stores, processes that information and execute the orders to control tissues that are essential to maintain energy homeostasis, controlling energy intake, energy expenditure and nutrient partitioning. Within the hypothalamus, the arcuate nucleus of the hypothalamus (ARC) is structurally unique with lacking blood brain barrier, and thus it receives direct signals (hormones, nutrients, amino acids, metabolites, etc) from the periphery. The ARC is mainly constituted by two neuronal populations, one leading to a positive energy balance express agouti-related protein (AgRP) and neuropeptide Y (NPY), while neurons leading to a negative energy balance express proopiomelanocortin (POMC). Our group has found that the lack of p53 in AgRP neurons, but not POMC neurons, causes that mice are more prone to develop diet-induced obesity. The reason for this is that these mice showed a late increase in food intake and especially because they had a reduced thermogenic activity in BAT (**Figure 1**). The mechanism modulating these actions involves the upregulation of MKK7 that activates c-Jun N-terminal kinase (JNK), an event that occurs previous to the development of obesity, and that was in agreement with a previous report indicating that activation of JNK signalling in AgRP neurons induced adiposity in mice. Supporting the important role of JNK, we also found that the pharmacological inhibition of central JNK reversed the obese phenotype of AgRP-Cre p53*^loxP/loxP^* mice. Furthermore, the knockdown of p53 in the ARC promoted weight gain in WT but not in JNK1-deficient mice. Although our results indicate that JNK is a key molecule for the regulation of central effects of p53, we must take into account that p53 is a transcription factor modulating the expression of a very large list of genes. Therefore, it is likely that p53 could modulate body weight through different mechanisms besides JNK.

**Figure 1:**
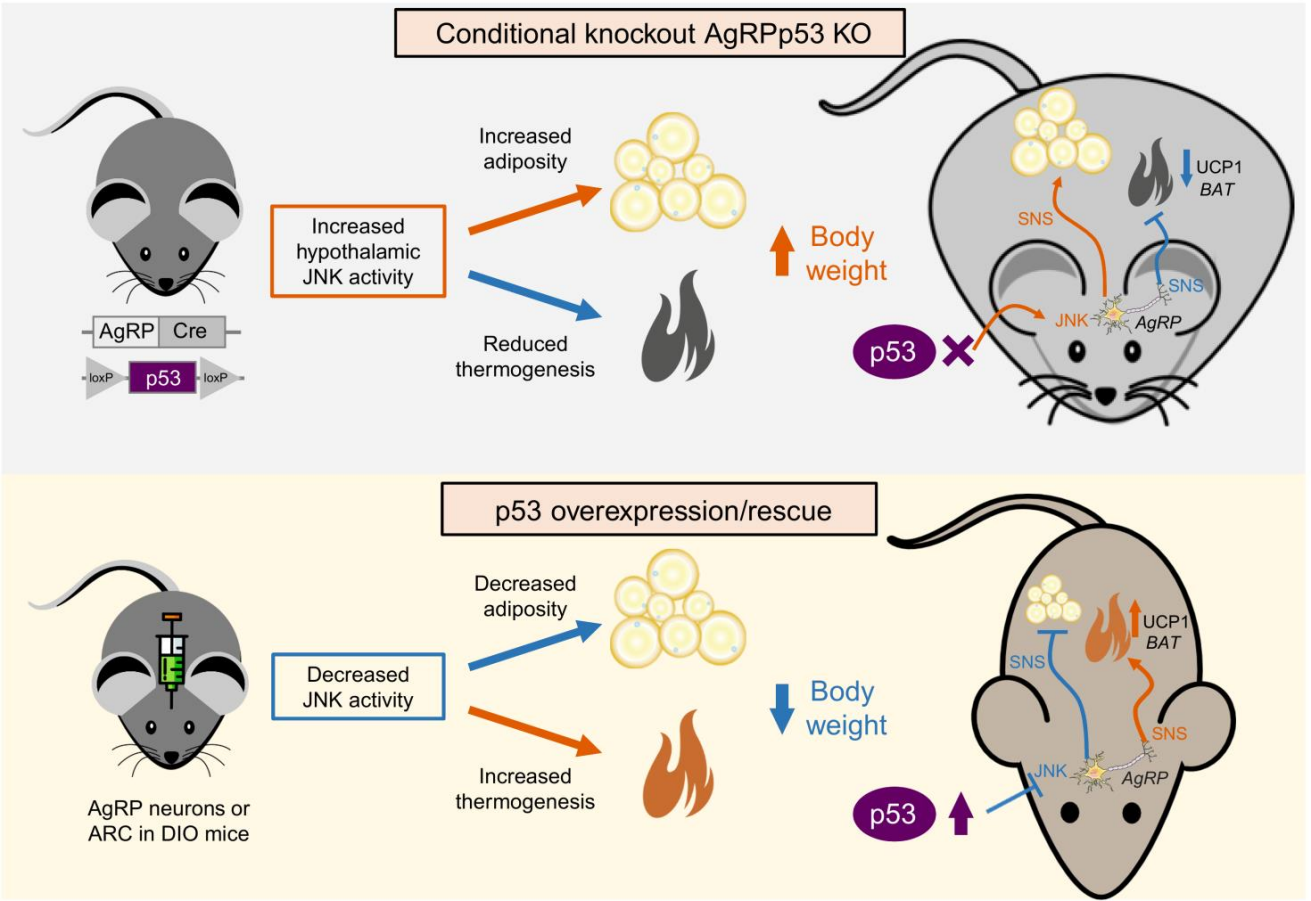
FIGURE 1: Schematic representation of the effects after ablation of p53 in AgRP neurons (upper panel) and the overexpression or rescue of p53 in AgRP neurons (lower panel).

Consistent with loss-of-function experiments causing weight gain, virogenetic tools overexpressing p53 in the ARC of diet-induced obese mice led to reduced weight gain by diminishing the levels of MKK4 and 7 and phosphorylated JNK, suppressed feeding and stimulated BAT thermogenic activity. Moreover, the recovery of p53 in AgRP neurons of conditional knockout mice ameliorated diet-induced obesity by triggering BAT thermogenesis. For exerting these effects on BAT, p53 requires an intact sympathetic nervous system, because the administration of an antagonist of the beta 3 adrenoreceptor, which mediates the activity of the sympathetic nervous system in adipose tissue, blunted brain p53-induced weight loss and BAT thermogenic activity.

Despite the metabolic function of p53 in the hypothalamus seems clear, an important question is whether these effects might be related or indirectly provoked by alterations in the integrity of AgRP neurons. In this sense, we failed to detect changes in markers of inflammation, apoptosis or senescence in the hypothalamus of mice lacking p53 in AgRP neurons, suggesting that metabolic effects can be dissected from those events. An interesting aspect to be studied in the near future will be whether hypothalamic p53 might regulate apoptosis and/or senescence at an advanced age, because our studies were done in young mice (maximum 18 week-old). Taking into account that mice lacking p53 globally need at least 16 weeks to develop tumors, it seems plausible to hypothesize that mice lacking p53 only in a particular neuronal subset would require a longer time to show alterations in several aspects related to cell cycle and viability. Another intriguing question is whether p53 may play an important role in extrahypothalamic areas that are also relevant in the control of energy balance like the brainstem, or brain regions related to food reward and motivation like the ventral tegmental area or nucleus accumbens. In this regard, it is worth to mention that peripheral signals like leptin (catabolic) or ghrelin (anabolic) not only act via hypothalamic neuronal pathways but also modulate hedonic mechanisms. Since we found that p53 in AgRP neurons modulated the orexigenic and adipogenic effects of ghrelin, it is tempting to speculate that perhaps p53 could also modulate the role of ghrelin -and other signals- in areas related to the hedonic control of food.

Although much is still unknown about the precise metabolic role of p53 in different organs, it is likely that future research will continue to unmask new exciting and unexpected functions of this transcription factor, that seems to be definitely much more than the guardian of the genome.

